# LightAnomalyNet: A Lightweight Framework for Efficient Abnormal Behavior Detection

**DOI:** 10.3390/s21248501

**Published:** 2021-12-20

**Authors:** Abid Mehmood

**Affiliations:** Department of Management Information Systems, College of Business Administration, King Faisal University, Al-Ahsa 31982, Saudi Arabia; aafzal@kfu.edu.sa

**Keywords:** anomaly detection, behavior analysis, fall detection, violence detection, suspicious behavior detection, convolutional neural network

## Abstract

The continuous development of intelligent video surveillance systems has increased the demand for enhanced vision-based methods of automated detection of anomalies within various behaviors found in video scenes. Several methods have appeared in the literature that detect different anomalies by using the details of motion features associated with different actions. To enable the efficient detection of anomalies, alongside characterizing the specificities involved in features related to each behavior, the model complexity leading to computational expense must be reduced. This paper provides a lightweight framework (LightAnomalyNet) comprising a convolutional neural network (CNN) that is trained using input frames obtained by a computationally cost-effective method. The proposed framework effectively represents and differentiates between normal and abnormal events. In particular, this work defines human falls, some kinds of suspicious behavior, and violent acts as abnormal activities, and discriminates them from other (normal) activities in surveillance videos. Experiments on public datasets show that LightAnomalyNet yields better performance comparative to the existing methods in terms of classification accuracy and input frames generation.

## 1. Introduction

As a part of continuously strengthening video surveillance systems, the automated detection of abnormal behaviors is becoming more relevant [1,2]. The need for improved techniques of autonomous detection is gaining more and more focus, mainly because of enormous amounts of surveillance data being generated and the impracticality of its manual monitoring because of the human toil involved. Several traditional (e.g., [3,4,5]) as well as deep learning-based methods (e.g., [6,7,8]) have focused on the problem. Abnormal events detection encompasses two types of video scenes: crowded and uncrowded [9]. The detection of anomalies in crowded scenes involves observing global motion patterns and events deviating from a normal behavior, e.g., sudden evacuation of everyone at the scene in an emergency. In contrast, movement patterns of individuals within an uncrowded scene are distinct and must be recognized in detail in order for them to be classified as abnormal. Some examples of abnormal behaviors at an uncrowded scene include falling, loitering, suspicious behavior (e.g., loitering, being in the wrong place (intrusions), a strange action that deviates from the learned behavior), and violence. There are two major difficulties in developing efficient abnormal behavior detection systems, and the existing literature has provided several methods to address both problems. First, there are difficulties inherent in the problem’s essence, i.e., recognizing the specifics of various (fundamentally different) behaviors. The challenge is further augmented because behaviors resemble each other in more than one way. So, for example, the acts of balancing attempts made by a person falling have much in common with the patterns commonly found in suspicious and violent behaviors. Therefore, the solutions targeting this problem mostly aim at providing inclusive methods for detecting multiple anomalies [3,10,11], customizing datasets to learn specific features of targeted behaviors [10], and using advanced techniques of learning the motion patterns [12,13,14], often by incorporating both spatial and temporal features. The second difficulty involves the computational complexity of behavior representation and detection algorithms, resulting in the high expense of computing resources, thus impeding their utilization in many real-world scenarios. To achieve the required efficiency that may enable real-time detection, the complexity of the model must be reduced. Therefore, many existing methods have aimed at reducing the complexity in various ways, such as by cascading local and global descriptors [15], using combinations of low complexity features instead of semantic features [16], and using spatiotemporal auto-encoder networks to extract abnormal behaviors [17]. It should be noted that a major contributing factor to the computational cost of video recognition systems is the underlying mechanism used for motion representation. Consequently, several proposals (e.g., [14,18,19]) have aimed at enhancing the recognition efficiency by focusing on computationally efficient methods representing temporal information. Furthermore, the architectural complexity of the learning models also affects the computational cost of recognition systems. Kim et al. [14] provided an interesting approach that, on one hand, encodes the temporal information efficiently, and on the other hand, eliminates the need for training highly complex 3D convolutional neural networks (3D CNNs) on large video datasets. In particular, they proposed the stacked grayscale 3-channel image (SG3I) format [14] that contains reasonably rich motion information with reduced computational expense, as compared with the same involved in other approaches like optical flow [20]. Later, they use a two-stream 2D architecture pre-trained on image datasets to learn the motion features for behavior recognition.

The current paper proposes a framework (termed LightAnomalyNet) that adopts the aforementioned approach of encoding the temporal information, and augments it by using a lightweight CNN architecture to distinguish between normal and abnormal events in surveillance videos. The framework essentially deals with the detection of anomalies as a binary classification problem by identifying some specific behaviors, including human falls, a few types of suspicious behavior, and some violent acts as abnormal activities, and discriminating them from other (normal) activities in surveillance videos. In this way, the proposed framework aims to address the two difficulties mentioned previously. It enables an accurate classification of normal and abnormal behaviors found in an uncrowded scene by learning the features related to each behavior. The framework deals with the problem of computational expense in two ways: (i) it adopts the SG3I format to create a dataset of images, thus eliminating the need for expensive optical flow computations, and (ii) it provides an approach using a lightweight CNN architecture that, unlike complex CNNs such as C3D [21] or ResNet [22], trains capably on a small to medium-sized dataset. The proposed framework is aimed at supporting a system that can be extended to work in a way like a typical intelligent video monitoring system deployed on a computer with a camera and network connectivity. It is important to emphasize here that the current work has a limited scope, as mentioned previously. A practical implementation of abnormal behavior detection systems also involves exploring many other aspects, including a consideration of the person–environment interaction, such as those reported in [4,23] for a fall detection system. Once implemented, such a system can take a video file or a webcam video stream as input, detect the abnormal behaviors as trained, and generate multiple types of alerts. The following are the major contributions of this study.

- The paper reviews the recent proposals of abnormalities detection, and determines a common set of those found in uncrowded scenes in videos. Specifically, it focuses on scenes featuring only one or a few persons, involving actions related to falling, suspicious behavior (e.g., loitering, being in the wrong place (intrusions), a strange action that deviates from the learned behavior), or violence.
- By focusing on more than one anomaly, as mentioned above, the study combines the classification of various commonly found abnormal behaviors in an uncrowded scene. As reported by previous studies such as [10], because of the challenges resulting from the essential similarities in the acts of various abnormal behaviors, the efficient joint detection of different anomalies (leading to an accurate classification as normal and abnormal behavior) is an interesting problem, and hence a notable contribution of the current study.
- A dataset based on SG3I image format is provided by selecting videos from publicly available datasets that are suitable for learning the behaviors involved in the study.
- The framework uses an advanced deep learning architecture that employs an effective method of motion representation, thus avoiding the use of expensive optical flow.
- We use a lightweight CNN architecture that effectively learns to classify the anomalies with high accuracy at low computational cost.

In the rest of this paper, we outline related studies in the next section. Section 3 discusses the details of the LightAnomalyNet framework. Section 4 provides a discussion of the experiments and an evaluation of the framework. Section 5 concludes the paper.

## 2. Related Work

Abnormal behavior detection is associated with the broader context of human action recognition and classification. Many studies in this background have extended the classical architectures such as [24,25] to further enhance the performance. To this end, Dai et al. [26] fine-tuned ResNet models pre-trained in Kinetics dataset on the UCF-101, and extracted the spatio-temporal features from video clips. Optical flow graphs obtained from the UCF-101 dataset were passed as input to optical stream to obtain optical features. Finally, a combination of both features was used for classification. We give some examples of the recent works in this area in the following. Ramya and Rajeswari [27] proposed an approach centered on the distance transform and entropy features extracted from images of human silhouettes obtained by subtracting the background. These features containing shape and local variation information are input to deep networks to classify human actions. Enhancing the efficiency of action recognition has been focused in many ways. Afza et al. [28] provided a framework that fuses and selects the most relevant features in order to enhance its computational and recognition performance. The method comprises four significant steps of frames enhancement, motion features extraction, length control fusion, and best feature selection, leading to efficient action recognition on selected datasets. Several techniques, such as discarding redundant features, extracting segments of interests, and feature descriptor mining, were adopted to improve the efficiency of human action recognition in uncontrolled environments in [29]. In a similar work [30], a reduction scheme was used to improve computational time and the accuracy of action recognition. Khan et al. [31] employed the fusion of segmented frames followed by implementing an entropy-skewness-based features reduction technique to obtain distinguishing features. Rashid et al. [32] proposed an object classification method that is based on multi-layer deep features fusion and selection. The fusion of features using the proposed technique and the selection of robust features have positively affected the computational time and classification accuracy. Another study [33] selects robust features by fusing three feature categories based on their highest values, and then using specialized methods to obtain most optimal features. Recently, Tsai et al. [34] proposed a deep learning-based system to recognize multiple concurrent actions performed by more than one person. They combined various algorithms to perform the essentials, such as locating individuals in the scene, tracking them, and recognizing them. An inflated 3D CNN (I3D) [35] was extended for action recognition.

Another area that is closely related to work in this study involves the detection of abnormal behaviors or events in a crowded scene. Ionescu et al. [36] formalized the crowd abnormal event detection as a one-versus-rest binary classification problem. They used object-centric convolutional auto-encoders that learn motion and appearance information. Each cluster of the training samples contained a specific normality. A binary classifier was then trained by distinguishing the positively labeled data points in a cluster from negatively labeled samples in all other clusters. Similarly, Smeureanu et al. [37] detected abnormal events by building and training a normality model using a one-class SVM classifier. At test time, they labeled the outliers detected by the approach as abnormal events. Zhang et al. [38] used the change of energy-level distribution to detect abnormal crowd behavior. Their approach treats image pixels as particles, and uses optical flow to obtain the velocities of those particles. They segmented the crowd motion based on flow field texture representation, and analyzed it based on changes in descriptors for the energy-level co-occurrence matrix. In a previous work, the author also proposed an approach [39] for crowd abnormal behavior detection by considering global abnormal events. That approach aimed at improving the overall efficiency by adopting a lighter form of a pre-trained 2D CNN for motion information. The model was trained on videos from crowd datasets, with high occlusion common for crowded scenes. The current work is essentially different from the previous work, since it focuses on detecting anomalies in scenes containing one or a few individuals (uncrowded) by learning specifics of actions. As the motion patterns of individuals in uncrowded videos are generally discrete, the current work has relied on using a lightweight model structure trained on videos containing uncrowded scenes. The combination of an effective motion representation technique with the lightweight structure has resulted in significant performance gains.

In addition, there are other wider contexts of anomaly detection. For example, Bakalos et al. [40] proposed an approach to detect abnormalities involved in various forms of attacks on water infrastructure. They proposed a framework based on multimodal data fusion and adaptive deep learning for the purpose. In the following, this section reviews the recent research aimed at providing efficient methods for autonomous monitoring systems for abnormal events in the specific context of uncrowded video scenes. A summary of the state-of-the-art anomaly detection methods for uncrowded scenes is provided in Table 1.

### 2.1. Traditional Methods

Several existing traditional methods have focused on fall events detection. Harari et al. [4] used the accelerometer and gyroscope sensors’ data collected by a smartphone to train a fall detection model. The detection was carried out by a continuous screening for the pre-defined acceleration threshold, followed by classification using a logistic regression model pre-trained in a dataset of simulated actions of falling. Vishnu et al. [5] developed a high-dimensional representation of falls and non-falls based on a fall motion mixture model that implicitly captures the motion attributes of each act. A low dimensional representation containing the attributes of abnormal actions for a specific video is extracted by performing factor analysis on the model. The method efficiently identifies falls in various scenarios. Min and Moon [41] detected falls found within a streaming video. They used an attended memory reference network to learn the features of the ongoing action by connecting the past information and visual memory pertaining to the action. A dedicated unit within the network detects the current action by referencing the visual information at each step. Zerrouki and Houacine [42] proposed a method for detecting falls by first characterizing the human body using curvelet transforms and area ratios features. To identify the posture, they adopted an SVM classifier and applied a hidden Markov model to distinguish fall events from other activities.

Some solutions in this category combined the detection of more than one abnormal behavior. Cheoi [3] proposed a method to detect various types of suspicious behaviors, including falling, suspicious (sudden) running, and violence in real-time, based on the underlying idea of detecting sudden changes in the magnitude and direction of motion. They used optical flow to determine distinct motion vectors for magnitude and direction of motion, which are processed to obtain a temporal saliency map. The regions with strong reactivity are recognized as abnormal. Kim et al. [11] merged various algorithms of detecting and tracking objects with those for the analysis of abnormal behaviors to provide a method to detect behaviors such as falling, loitering, violence, and intrusion, based on the surveillance of pedestrians.

### 2.2. Deep Learning-Based Methods

Many CNN-based methods have been proposed to detect falls. Nunez et al. [43] used a CNN that extracts motion features from optical flow images to identify falls from non-falls. Specifically, they adopted a VGG-16 architecture that is first trained on the ImageNet dataset from scratch, and then fine-tuned on the optical stacks of UCF-101. Later, transfer learning is applied to fine-tune the network on three datasets specific to fall events. Yao et al. [6] also provided a fall detection system by adopting geometric features for training a CNN. They obtained the geometric features by segmenting the head and torso using the traditional ellipse fitting method, and they employed the same information to extract motion features. Next, they used a shallow CNN structure to learn the motion features. Khraief et al. [44] used a multi-stream CNN comprising four streams to detect falls using multimodal data captured by RGB-D cameras. Each stream of the CNN dealt with a distinct modality. Specifically, by combining various modalities, including RGB and depth images, they could deal distinctly with static appearance, shape variations and motion information, and achieve higher classification accuracy.

Most of the recent methods of abnormal behavior detection focus specifically on detecting violence. Pan et al. [7] used a two-stream inflated 3D CNN (inception-v1) to work on spatial and temporal (optical flow) information, to extract the features from video streams. The features extracted from two streams of the network are fused and passed to a GRNN classifier, which replaces the softmax classification layer of the original i3D model, for making predictions. For violence detection, the model achieved high accuracy on a UCFCrime dataset. Roman and Chavez [45] proposed a semi-supervised method that, besides detecting violence, also aims to address the problem of the lack of violence datasets with spatial annotations. For violence detection, they summarized the video sequences into dynamic images [53] and used these images to train a CNN classifier. Rendón-Segador et al. [8] adopted a 3D DenseNet and combined it with a self-attention mechanism, and a bidirectional convolutional LSTM, to detect violence. The method relies on the optical flow as input, which is first encoded by the DenseNet into sequences of feature maps, and then passed on to self-attention and ConvLSTM layers before carrying out prediction by the fully connected layers of the classifier. Ullah et al. [46] analyzed the sequential patterns found in surveillance videos to develop a method for violence detection in industrial video stream. This method addressed resource expense by preprocessing the video stream to select the most informative shots. It encoded the dynamics related to various actions involved in violence using optical flow features. An LSTM network finally learned and classified the violent activity patterns over a period. Asad et al. [13] adopted a multi-level feature fusion approach to integrate local motion patterns from an equally spaced sequence of input frames. They combined a wide-dense residual block with a 2D-CNN to learn combined features obtained from pairs of input frames. LSTM units lastly captured temporal dependencies. The model yielded high accuracy in four datasets of violent behaviors. Ullah et al. [47] adopted a pre-trained ResNet-50 architecture to extract the spatio-temporal features, and passed them on to a multi-layer bidirectional LSTM model to classify anomalies in surveillance videos. Ullah et al. [48] exploited the one-shot learning strategy for anomaly recognition to develop a method for violence detection. The method adopted a lightweight Siamese 3D CNN on the underlying principle of learning the similarities between shots, and efficiently classified the anomalies based on the dissimilarities between two given sequences. Similarly, a 3D CNN was implemented in [49] to sample the key frames based on a gray centroid before passing them for classification.

Some methods have addressed the detection of various types of suspicious behaviors. Since these behaviors are of many types, each method has focused on a specific subset pertaining to a context. Fang et al. [50] used a modified form of the YOLOv3 algorithm to detect abnormal behaviors commonly observed during an examination. For this purpose, the authors also produced a video dataset of common violations in an exam setting, such as a person bending over the desk or placing a hand under the table. Sha et al. [51] detected five different behaviors (including two abnormal behaviors) in a specific industrial setting. They adopted a two-stream DenseNet to extract spatial and temporal features from a self-collected dataset. Chriki et al. [52] have proposed a method for surveillance with the help of unmanned aerial vehicles (UAVs). It combined the use of CNN with hand-crafted methods (HOG and HOG3D) for feature extraction. It carried out the classification of different abnormal behaviors using a one class SVM. The method could accurately classify different suspicious behaviors found in the mini-drone video dataset. Mehmood [10] studied the specifics of motion patterns involved in three abnormal behaviors, i.e., falling, loitering, and violence, and developed a new dataset by selecting videos pertaining to those patterns from public datasets. A two-stream inflated 3D CNN model pre-trained on the Kinetics dataset was then fine-tuned on the newly developed dataset for the detection of the three anomalies. This work is closely related to the current study in the sense that it aims at detecting different abnormal behaviors in uncrowded scenes. However, it works on a dataset created by selecting videos from public datasets related to each of the three abnormal behaviors detected by the study. It conducted the performance evaluations based on the customized dataset, instead of the original public datasets. The current study trains and evaluates the model on public datasets directly. Besides, instead of using optical flow and a 3D network, the current study uses a more optimized form of both the motion representation and the network, as detailed in the next section.

## 3. The Proposed Framework

The key goal of this work is to provide an efficient framework for detecting anomalies in behaviors found in uncrowded video scenes. As efficiency is one of the key design goals of the proposed framework, we must specify the context of the study and the way it aims at dealing with improving the detection efficiency. The main idea is to develop a general-purpose technique that can be further customized and deployed in a variety of environments. In this way, our proposal essentially resembles many existing approaches of abnormality detection detailed in Section 2. As far as the efficiency is concerned, we consider a specific perspective of reducing the computational complexity and memory space requirements. In other words, our objective is to propose an approach that accurately classifies the abnormal behaviors while reducing the number of computational operations (such as convolution, pooling, batch normalization, and activations) and the amount of memory required to run the system—the two key factors affecting the computational complexity of deep learning-based systems. We aim to achieve better results comparative to existing approaches in this context. A system that achieves better results along these lines can be adapted for a variety of environments, such as large-scale surveillance in distributed environments [54]. However, further investigation of the suitability of the approach in each environment was deemed out of the scope of the current study.

Here, it will also be worthwhile to elaborate on the specific ways in which the current study attempts to achieve the aforementioned target of reducing the computational complexity and memory space requirements. Video data used by an action recognition system include both spatial and temporal information. The frames extracted from a video can directly serve as a source to learn spatial objects. In addition, some other mechanism is required to absorb the motion information found in the sequential frames. The typical methods used for learning the motion information, such as optical flow [20] and dynamic images [53], require a great deal of computational load and memory space [53,55,56], which limits their use in real problems [56], and thus identifying the alternatives is an open research area. Over the years, many attempts have been made along these lines. For example, 3D CNNs have been trained by directly feeding the video sequence [21,57,58,59,60]. Yet, because of the complex 3D convolutions, 3D CNNs need an exceedingly high number of computations and memory space. A pseudo-3D (P3D) CNN [61] was also proposed to minimize the effect of 3D convolutions. However, it reduces the size of a 3D CNN by only a limited factor, and is still heavy [61]. We intend to address the problem with a different technique.

**Figure 1:**
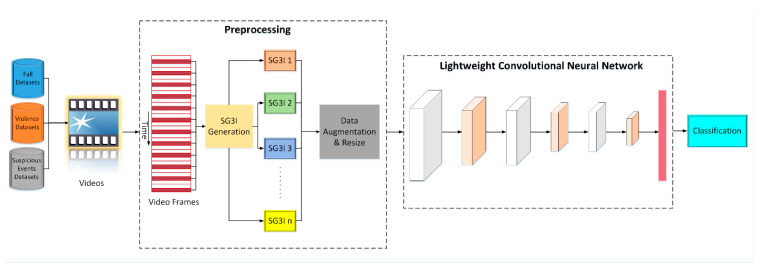
The overall architecture of the proposed LightAnomalyNet framework.

The proposed LightAnomalyNet achieves detection efficiency via two principal components. First, rather than relying on methods that demand huge computational loads and memory space such as using optical flow frames in two-stream network (similar to methods of [13,43]) or feeding the video directly to 3D CNN (similar to methods of [58,60]); it adopts a low computational cost method of modeling motion features, i.e., the stacked grayscale 3-channel image (SG3I) of Kim and Won [14]. In this way, it is expected that SG3Is will enable capturing of motion details effectively because of the low occlusion in uncrowded scenes and the existence of explicit actions. Second, instead of using highly complex neural network architectures for training and classification, LightAnomalyNet uses a lightweight network structure inspired from [62] that is simple enough to minimize the computational loads, but can provide high accuracy when trained on SG3I images. This latter characteristic of the proposed framework also relieves it from the requirement of a large video dataset or a pre-trained network, simply because the simple CNN (unlike complex alternatives such as C3D or i3D) can be trained well on a small-to-medium-sized dataset. An overall view of the proposed LightAnomalyNet framework is shown in Figure 1. A detailed discussion of the process comprising the method for generating SG3I images followed by their classification into those containing normal and abnormal behaviors using the lightweight CNN is provided in the following subsections.

### 3.1. Input Images Generation

The proposed framework for anomaly detection uses a lightweight structure of a 2D CNN in order to avoid the computational cost involved in training 3D architectures. Here, it must be ensured that the network is trained with an input format that enables it to learn the motion features found in videos efficiently. Therefore, owing to its ability to detect motion effectively while reducing the computational cost involved in methods such as optical flow, the stacked grayscale 3-channel image (SG3I) format [14] is used to capture the motion features pertaining to both normal and abnormal actions.

**Figure 2:**
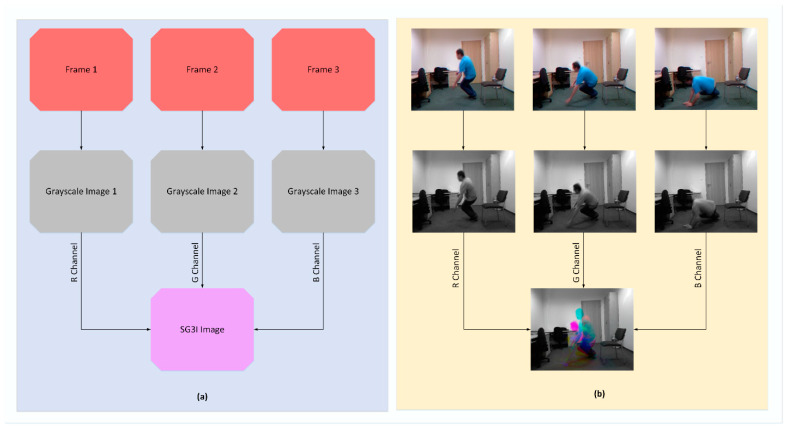
(**a**) Process of generating SG3I images from sequential video frames; (**b**) Example of SG3I generation for URFD dataset.

**Figure 3:**
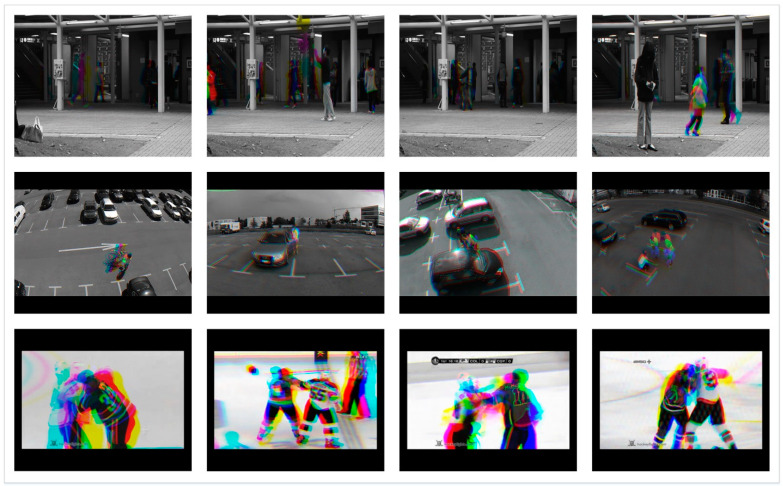
Sample SG3I images generated for Avenue (row 1), Mini-Drone Video (row 2), and Hockey Fights (row 3) datasets.

As far as the conversion to SG3I format is concerned, it is a two-step process that takes three sequential frames from a video and outputs a single 3-channel RGB image, as illustrated in Figure 2 with an example. In the first step, it converts each of the three frames into a grayscale image, thus producing three grayscale images; let us call them gs1, gs2, and gs3. In the second step, the grayscale images gs1, gs2, and gs3 are incorporated into R, G, and B channels, respectively, of a new single-color image of SG3I format. Now, before looking at the details of motion representation in SG3Is, let us recall that, for three identical images, the RGB values for each pixel remain the same. For three sequential frames involving some motion, the RGB values for each pixel representing the moving object differ for each of the frames. This means that the pixels with an identical value for RGB channels providing a grayscale output in the SG3I image will represent the static regions of the frame. Meanwhile, the pixels with difference in RGB values across the three frames, thus resulting in a color or displacement in brightness, will show the moving objects in the frame. Therefore, the colored regions (i.e., hue) within the SG3I are expected to characterize the motion patterns effectively. There is one detail that must be noted here. Since the SG3I image encodes motion information from three consecutive frames, the selection of these frames must be carried out in an optimized way. We must keep the uniform time interval between consecutive frames short enough to make the SG3Is insensitive to noisy motions such as camera movement. At the same time, we must not make the time interval so short that it prevents the SG3Is from capturing meaningful information about the motion taking place in the consecutive frames. Therefore, a balance was accomplished by adopting a technique similar to [14]. Specifically, the technique divides the video clip into many sub-clips of a configurable size, followed by generating one SG3I for each sub-clip. In this way, it is also ensured that the selected frames are representatives of all segments of the entire video. Sample SG3Is obtained from three datasets adopted in this study are shown in Figure 3.

### 3.2. CNN Model Architecture

**Figure 4:**
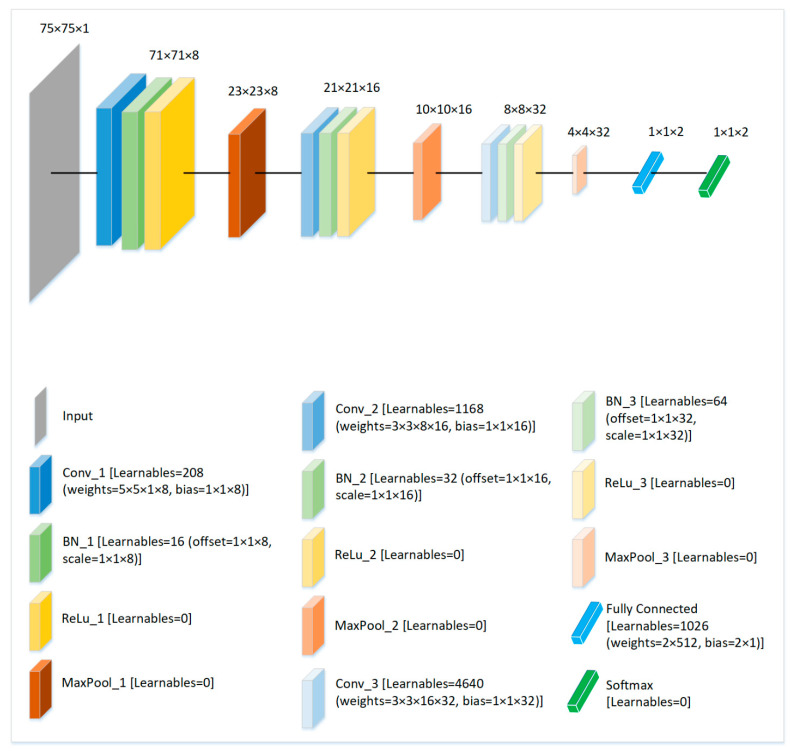
Proposed architecture of the lightweight CNN and the analysis of learnable parameters at each layer of the network. Note that the total of all learnable parameters for the proposed structure of the CNN is 7154.

The LightAnomalyNet framework proposes a lightweight CNN architecture, with a minimal number of activation resolutions and learnable parameters, instead of using deeper architectures. The primary motivation behind adopting a lightweight architecture is to achieve computational efficiency, while avoiding possible over-fitting. Therefore, several lightweight CNN architectures were thoroughly experimented with SG3Is. In particular, various options of the number of convolutional layers, channels, filter sizes, and pooling layers were evaluated during the architecture development. Finally, it was observed that the proposed CNN architecture inspired from [62] (see Figure 4) works best with the SG3Is for the anomaly detection problem in resource constrained applications. Note that SG3Is were also experimented with other recognized deep networks for the purpose, and the results will be presented in Section 4.2.

Before passing to the lightweight CNN, the input SG3Is are resized to 75 × 75 by cropping a patch of this size around the pixels containing motion (specifically, color or hue, which is determined by the difference of values among RGB channels in the same pixel of SG3I). This step of resizing is carried out so that only the part containing potentially higher amount of useful information can be preserved. A reduction in the spatial dimensions by a technique that is considerate of the informative pixels allows the use of a simplified model with fewer network parameters without affecting the accuracy. As shown in Figure 4, following the input layer, the proposed CNN comprises 3 sets of a 3-layer structure, each containing convolutional, batch normalization, and ReLu layers. The 3-layer structures are separated by respective max pooling layers. In this way, the first 3-layer structure contains a convolutional layer that uses 5×5 filters with 8 channels, followed by a batch normalization, and a ReLu activation layer. A 3×3 max pooling operation follows the first structure. Furthermore, apart from the batch normalization and ReLU layers, the second 3-layer structure contains the convolutional layer with 3×3 filters and 16 channels and is followed by a 2×2 max pooling. Similarly, the convolutional layer within the third structure uses 3×3 filters with 32 channels and the structure is followed by a 2×2 max pooling. Finally, a fully connected layer comprising two nodes is applied, and the softmax is employed for predictions. An analysis of the proposed CNN architecture containing the details of activation resolutions and learnable parameters is also presented in Figure 4. Note that the low number of total learnable parameters (7154) is a distinguishing characteristic of the proposed network, as we will see in comparison with the other popular networks in Section 4.2.

### 3.3. Model Training and Testing

To train and test the model on the designated datasets, the techniques described in Section 3.1 were applied to obtain SG3I images from video clips. For each of the training and testing phases, the SG3I images were generated for the corresponding split of each of the datasets. More details of the train/test splits of the datasets are provided in Section 4.1. Furthermore, data augmentation is a standard technique to deal with class imbalance and the lack of data for training [63]. We performed data augmentation with a goal to increase the generalizability of the model. While the model constantly sees new, slightly changed versions of the inputs, there is a greater possibility that it learns more robust data patterns quickly. For data augmentation, a random cropping to the center was performed on the originally obtained SG3Is that were followed by a horizontal flip, and finally resizing into dimension 75×75. During training, we analyzed the model performance with two different optimizers, i.e., stochastic gradient decent (SGD) and Adam. For each of these optimizers, different learning rates, momentum, weight decay, and nesterov acceleration parameters were experimented. To this end, after setting a learning rate, we followed a general approach of reducing it by 1/10 whenever there was no loss reduction for over 10 epochs. The best results were observed with the Adam optimizer when used with a learning rate of 0.001, beta1 value of 0.9, beta2 value of 0.999, epsilon value of $1\times 10^{-8}$, and amsgrad value of false. The same network configuration was used consistently for all datasets during the testing phase, and for the experiments presented in the next section.

## 4. Experiments

The proposed LightAnomalyNet was thoroughly evaluated experimentally to measure its strength in detecting abnormal behaviors in uncrowded scenes and to compare it with the existing methods in the literature. The entire system was implemented in Python and Keras with TensorFlow 2.0 in an Ubuntu 20.04 environment. The experiments involving the proposed model and SG3Is were conducted using a CPU-only configuration (Intel i7-8650 @2.11 GHz 32 GB RAM). As detailed later in Section 4.2, some experiments conducted for comparison required optical flow computations, for which a GPU (NVIDIA GTX 1080Ti 11 GB, Santa Clara, CA, USA) setting was used.

### 4.1. Datasets

Four public datasets closely related to the behaviors involved in the current study, i.e., UR Fall [64], Avenue [65], Mini-Drone Video [66], and Hockey Fights [67] datasets were adopted to evaluate the proposed framework of anomaly detection. A summary of the statistical information of the datasets adopted for SG3Is formation is given in Table 2.

**UR Fall dataset:** The UR Fall dataset [64] contains a total of 70 videos (30 falls and 40 not-falls) with a resolution of 640×480. Fall events are recorded from two different perspectives with separate cameras, whereas only one camera was used for the other events. Each video contains a single actor performing the activity. For the training and testing of our model, we used the videos obtained from the same perspective (camera 0) for both types of events. Since the dataset documentation does not explain the train/test splits, the videos were divided into three groups. Each group contains 16 videos (8 for each of the fall and not-fall categories). Testing was carried out using a separate split of 12 videos.

**Avenue dataset:** The Avenue dataset comprises 16 training and 21 testing videos with a resolution of 640×360. The videos are captured on a CUHK campus avenue. The training set contains videos that capture normal situations only, whereas the videos in the test set include both normal and abnormal events. The abnormal behaviors include sudden running, holding an abnormal object, loitering, entering a group of people from the opposite direction, and other actions that would draw the attention of the surveillance staff. To enable the model’s learning of the features related to both normal and abnormal behaviors, we divided the videos into 3 groups each containing 13, 12, and 12 videos, respectively, in a way that each group contained a judicious mix of videos from both sets. Furthermore, a consistent approach for the train/test splits was adopted. In particular, while testing a video from each group, all videos except the one being tested were used to train the model.

**Mini-Drone Video dataset:** The Mini-Drone Video dataset contains 15 training and 23 testing HD videos of resolution 1920×1080 with a Phantom 2 Vision+ in a car parking. To optimize for minimizing the unnecessary processing load while maintaining quality, the videos were initially converted to a resolution of 640×480. Both the training and testing sets contain various videos containing normal and abnormal scenes. Here, the anomalies include people engaging in suspicious activities such as loitering around parked cars, and other abnormal actions such as mis-parking their cars, stealing, and other activities that would attract the interest of the surveillance staff. To create an assortment of videos containing various abnormal behaviors, we redistributed the 38 videos into 3 subsets of 13, 13, and 12 videos each. Each subgroup contains videos related to normal and abnormal behaviors. As far as the train/test splits are concerned, the same approach was used as described above for the Avenue dataset. So, in order to test a video, only the video being tested was left out, and all the remaining videos in the subset were used for training.

**Hockey Fights dataset**: The Hockey Fights dataset contains two groups (fights and non-fights) of a total of 1000 videos of resolution 360×280. The videos were shot from different angles and contain normal and violent activities occurring in both crowded and uncrowded scenes. To fulfill the requirements of the current study, 210 uncrowded video clips (involving 2–3 players) were selected. The clips were then divided into 3 groups, each containing 35 videos from the fights and 35 from the non-fights classes. A separate split of 20 videos was created for testing.

### 4.2. Overall Performance Evaluation

**Figure 5:**
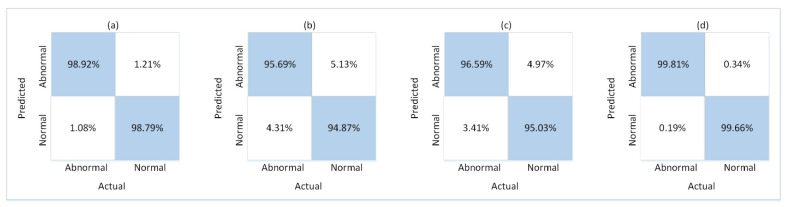
Confusion matrix of the proposed framework on: (**a**) UR Fall dataset (**b**) Avenue dataset (**c**) Mini-Drone Video dataset (**d**) Hockey Fights dataset.

**Figure 6:**
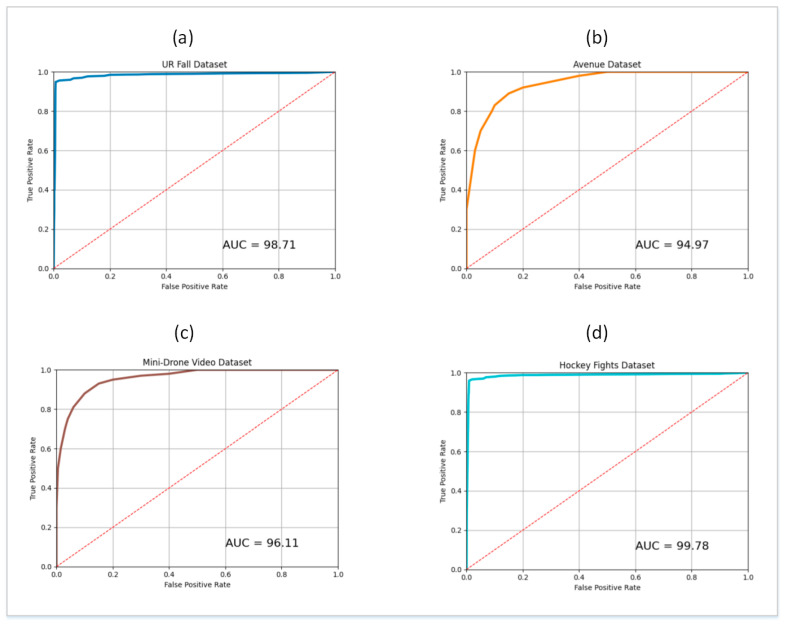
ROC curve and AUC values for: (**a**) UR Fall dataset (**b**) Avenue dataset (**c**) Mini-Drone Video dataset (**d**) Hockey Fights dataset.

During experiments, the train and test splits of the four datasets described in Section 4.1 were used for the respective phases of training and testing. For this purpose, 10 SG3I images per video clip were used to train the model, and 10 SG3Is to test it. The SG3Is were sampled using the techniques described in Section 3.1. The number of SG3I images to be used for training and testing was adopted from [14], wherein the authors of SG3I reported the best performance results using 10 SG3I images in the temporal stream (see Table 1 in [14]). So, the performance of the proposed lightweight model with SG3Is was evaluated using the test split of each dataset. It was measured in terms of the number of correct predictions made for abnormal as well as normal classes. Figure 5 presents the confusion matrix showing the results of model performance against both classes. The proposed framework correctly classified 98.92%, 95.69%, 96.59%, and 99.81% of the abnormal test cases from UR Fall, Avenue, Mini-Drone Video, and Hockey Fights datasets, respectively. Similarly, it accurately classified 98.79%, 94.87%, 95.03%, 99.66% of the normal test cases from UR Fall, Avenue, Mini-Drone Video, and Hockey Fights datasets, respectively. We notice that the percentage of normal behavior mis-classified as anomaly is a little higher than that of anomalies mis-classified as normal behavior for all four datasets. However, it was deemed satisfactory, since having a slightly higher number of false positives is more acceptable than a higher false negative rate for an anomaly detection system. By looking at the results on different datasets individually, one can see that the model’s misclassification rates are significantly higher for Avenue and Mini-Drone Video datasets comparative to the other two datasets. This was attentively investigated during experiments to exclude any potential erroneous performance of the model. However, it was concluded that the misclassifications can be attributed to the extent of variation observed in the actions found in the datasets and the scene complexity. The two datasets with higher misclassification rates contain scenes with much more complexity and diversity as compared to the UR Fall and Hockey Fights datasets. Furthermore, the ROC curves and AUC values for each dataset shown in Figure 6 provide more insight into the true positive and false positive rates of the model. The AUC values of the model on UR Fall, Avenue, Mini-Drone Video, and Hockey Fights datasets are 98.71, 94.97, 96.11, and 99.78, respectively.

**Figure 7:**
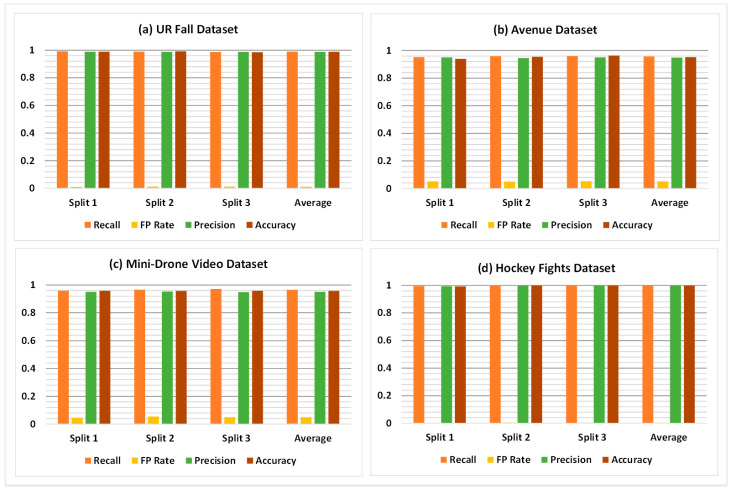
Comparison of classification results on different splits of: (**a**) UR Fall dataset (**b**) Avenue dataset (**c**) Mini-Drone Video dataset (**d**) Hockey Fights dataset.

The overall classification results of the proposed framework for all four datasets are shown in Table 3. The results indicate that the model exhibits the ability to generalize well for the variety of abnormal behaviors found in the datasets. The generalization ability enables the model to distinguish accurately between unique events and yields adequate classification performance. Specifically, the F_1_ score, which is a combination of recall and precision of a model, is 98.86%, 95.30%, 95.84%, and 99.74% on UR Fall, Avenue, Mini-Drone Video, and Hockey Fights datasets, respectively. Table 4 shows the results of a detailed statistical analysis of the proposed framework for all four datasets. For this purpose, the accuracy of the proposed model was obtained for 100 iterations to give the minimum, average, and maximum values of accuracy. The standard deviation, standard error, and margin of error (MoE) values were computed and observed to further assess the performance of the classification model. Figure 7 shows a comparison of the performance results achieved with the model on different splits of the four datasets. It shows that the overall performance of the model remains nearly identical in different splits of each dataset.

### 4.3. Evaluation Based on Execution Time

As discussed in the introduction, the architectural complexity of the learning models significantly affects the computational cost of behavior recognition systems. Therefore, one motivation behind using SG3Is within LightAnomalyNet framework was to eliminate the need for expensive motion representation methods, such as optical flow [20] and dynamic images [53]. Therefore, it is imperative to verify the computational effectiveness of the use of SG3Is. We provide a comparison of the execution times (measured as frames per second or fps) for the three methods, i.e., optical flow, dynamic images, and SG3I, in Table 5. Note that this evaluation was carried out to substantiate the results shared by the SG3I paper [14], which reported results on UCF-101 and HMDB-51, on the datasets adopted by the current study. As shown in the table, despite the use of a GPU-based environment for obtaining and executing optical flows, the SG3I method generates input frames with much higher speed compared with the other two methods.

### 4.4. Comparison with Other Networks

As the dataset of SG3I images works with any pre-trained network, we also evaluated the performance of the combination of the proposed lightweight CNN and SG3Is compared to the other deep networks commonly used in the literature for abnormity detection. Specifically, we used a combination of SG3Is with each of the ResNet-50 [25], Inception-V3 [68], and DenseNet-250 [69], and compared the results. For these networks, transfer learning was performed by adjusting the input image sizes to match with those of each of the pre-trained network, and replacing the final layers of networks to output only two classes, i.e., normal and abnormal. The results are shown in Table 6. In general, all networks perform almost equally in terms of accuracy. As the deep architectures require large datasets for training, the proposed lightweight architecture works well with the existing commonly used anomaly detection datasets, while requiring an exceedingly low number of trainable weights (a total of 7154 weights, as detailed in Figure 4). It is important to recall here that this work aimed at reducing the number of computational operations and the amount of memory required to run the system. As shown in Table 6, the network latency (measured as time per inference step) and the model size are noticeably lower than other networks.

### 4.5. Comparison with the State-of-the-Art

The performance of the proposed framework for anomaly detection was also evaluated in comparison with the existing state-of-the-art works in the area. To this end, we selected the methods that have reported the highest performance in each of the three major categories addressed by this study, i.e., falling, suspicious actions, and violence. Note that, since researchers have presented the performance results using a variety of metrics, the comparative results in this section are shown using the metrics and datasets used in the original study. The results of comparison with methods in the falling category are presented in Table 7. The proposed framework outperforms the existing methods in UR Fall dataset by yielding an accuracy of 98.86% versus the preceding accuracies of 97.0% (Zerrouki and Houacine) and 95.0% (Nunez et al.). Nunez et al. and Khraief et al. reported better results in terms of recall (100.0%). However, the superior precision provided by the proposed framework (i.e., the fraction of predictions of falling that were actually falls) shows that it has a better prediction performance as compared with the state-of-the-art.

The results of comparison with approaches in the suspicious action category are tabulated in Table 8. For this category, LightAnomalyNet provides higher results in both Avenue and Mini-Drone Video datasets. Specifically, it yields an accuracy of 95.28% as compared with the accuracy of 90.1% reported by Cheoi on the Avenue dataset. Furthermore, the proposed framework is more accurate on Mini-Drone Video dataset (95.81% versus 93.57% of Chriki et al.). Table 9 presents a comparison of methods in the violence category. Here, the proposed LightAnomalyNet provides better results in the Hockey Fights dataset. Specifically, it achieves an accuracy of 99.74% on the dataset in comparison with the existing methods in the category, such as Roman and Chaves (96.40%), Song et al. (99.62%), Ullah et al. (98.00%), Asad et al. (98.80%), and Mehmood (99.71%). Hence, the results in all three categories of anomalies in the uncrowded scenes show that the proposed lightweight framework achieves better results than the existing methods of abnormality detection. The overall gains in accuracy comparative to the existing methods can be attributed to the following key factors. SG3I captures the patterns of motion and differences in actions effectively. This enables the model to learn the discriminative features well to distinguish between normal and abnormal actions. The learning is further augmented by supplying only the most relevant part of the SG3I, thus allowing the network to focus on the significant features. The combination of lightweight structure with SG3I has also contributed positively as determined by the results of comparison with other networks.

## 5. Conclusions

This paper presented a framework (called LightAnomalyNet) that uses a lightweight CNN architecture for detecting anomalies in the actions found in videos. The study addressed three categories of the abnormal behaviors that are commonly found in uncrowded scenes, i.e., falling, suspicious action, and violence. To achieve high classification performance while allowing for low computational costs, the proposed LightAnomalyNet adopts SG3Is (stacked grayscale 3-channel images) to train a lightweight CNN. When combined with the lightweight CNN structure, SG3Is provide a potent alternative to classical methods of motion representation, such as optical flow and dynamic images. The proposed framework achieves relatively better recognition performance and computation efficiency as compared to the existing methods. So far as classification accuracy is concerned, the experiments on UR Fall, Avenue, Mini-Drone Video, and Hockey Fights datasets show that the proposed framework can efficiently detect various anomalies found in these datasets with accuracies of 98.86%, 95.28%, 95.81%, and 99.74%, respectively.

## Figures and Tables

**Table 1:** Summary of the abnormal behavior detection methods.

Reference	Data Used	Feature/Model	Type(s) of Anomaly Detected	Dataset(s)
**Traditional Methods**				
Harari et al. [4]	Accelerometer data, gyroscope signals	Acceleration threshold, logistic regression-based classifier	Falling	Self-collected
Vishnu et al. [5]	RGB	GMM, FMMM, fall motion vector	Falling	UR Fall Detection, Montreal
Min and Moon [41]	RGB	Embedding module, attended memory module	Falling	AI Hub DS
Zerrouki and Houacine [42]	RGB	Curvelet transforms, area ratios features, SVM-HMM	Falling	UR Fall Detection
Cheoi [3]	Optical flow	Optical flow, temporal saliency map	Falling, violence, suspicious	UMN, Avenue, Self-collected from CCTV footage
Kim et al. [11]	RGB	Object detection, YOLOv4	Falling, intrusion, loitering, violence	Korea Internet & Security DS
**Deep Learning-Based Methods**				
Nunez et al. [43]	RGB, optical flow	2D-CNN	Falling	UR Fall Detection, Multicam, FDD
Yao et al. [6]	RGB	GMM, 2D-CNN	Falling	Self-collected
Khraief et al. [44]	RGB, depth images	Multi-stream CNN	Falling	Self-collected, UR Fall Detection, FDD
Pan et al. [7]	RGB, optical flow	3D-CNN	Violence	UCF-Crime, UCF-101
Roman and Chavez [45]	RGB	CNN	Violence	Hockey Fights, Violent Flows, UCFCrime2Local
Rendón-Segador et al. [8]	RGB, optical flow	Multi-head self-attention, bidirectional convolutional LSTM	Violence	Hockey Fights, Movies, Violent Flows, Real Life Violence Situations
Ullah et al. [46]	RGB, optical flow	CNN	Violence	Hockey Fights, Violent Flows, Surveillance Fight
Asad et al. [13]	RGB	Feature fusion, 2D-CNN, LSTM	Violence	Hockey Fights, Movies, Violent Flows, BEHAVE
Ullah et al. [47]	RGB	Spatiotemporal features, CNN, bidirectional convolutional LSTM	Violence	UCF-Crime, UCFCrime2Local
Ullah et al. [48]	RGB	3D-CNN	Violence	UCF-Crime
Song et al. [49]	RGB	Key frames sampling, 3D-CNN	Violence	Hocky Fights, Movies, Violent Flows
Fang et al. [50]	RGB	CNN, YOLOv3	Suspicious	Self-collected
Sha et al. [51]	RGB, optical flow	Two-stream 2D-CNN	Suspicious	Self-collected
Chriki et al. [52]	RGB	HOG, HOG3D, CNN	Suspicious	Mini-Drone Video Dataset
Mehmood [10]	RGB, optical flow	2-stream 3D-CNN	Falling, loitering, violence	UFLV

**Table 2:** Statistical Information of the datasets adopted for SG3Is preparation.

				Anomalous Samples			Non-Anomalous Samples		
Dataset	# Video Samples Used	Frame Rate	Resolution	# Samples	# Anomaly Sequences	# Frames	# Samples	# Non-Anomaly Sequences	# Frames
UR Fall *	48	30	640×480	24	24	720	24	250	7500
Avenue	37	25	640×360	18	57	3750	19	238	10,350
Mini-Drone Video	38	30	640×480	24	43	6380	10	24	2925
Hockey Fights **	70	25	360×280	35	35	875	35	35	875

* a separate set of 12 videos was used for testing. ** a separate set of 20 videos was used for testing.

**Table 3:** Classification results of the proposed framework on four datasets adopted in the study.

	UR Fall	Avenue	Mini-Drone Video	Hockey Fights
Recall	0.9892	0.9569	0.9659	0.9981
FP Rate	0.0121	0.0513	0.0497	0.0034
Precision	0.9879	0.9491	0.9511	0.9966
Accuracy	0.9886	0.9528	0.9581	0.9974
F_1_	0.9886	0.9530	0.9584	0.9974

**Table 4:** Statistical analysis of the proposed framework based on Margin of Error (MoE) at confidence level 95%.

Dataset	Accuracy (%)—100 Iterations			Statistical Measures		
	Minimum	Average	Maximum	Standard Deviation	Standard Error	MoE
UR fall	97.01	98.06	98.88	0.5574	0.0258	0.1098
Avenue	93.06	94.21	95.54	0.7432	0.0342	0.1464
Mini-drone video	93.09	94.24	95.83	0.7643	0.0435	0.1506
Hockey fights	98.76	99.34	99.92	0.3247	0.0147	0.0640

**Table 5:** Comparison of the execution times (frames per second) taken for input frames generation.

Dataset	Optical Flow	Dynamic Image	SG3I
UR fall	16.59	175.10	719.61
Avenue	15.93	184.65	776.12
Mini-drone video	16.09	189.14	789.36
Hockey fights	16.77	177.85	745.70

**Table 6:** Comparison of the proposed lightweight model with different networks.

Network	No. of Learnable Parameters	Size (MB)	Time per Inference Step (ms)—CPU	Time per Inference Step (ms)—GPU	UR Fall Accuracy%	Avenue Accuracy%	Mini-Drone Video Accuracy%	Hockey Fights Accuracy%
ResNet-50 + SG3I	25M+	106	698.40	45.50	97.92	95.78	95.18	99.78
Inception-V3 + SG3I	23M+	101	507.00	68.60	98.89	95.17	95.86	99.71
DenseNet-250 + SG3I	15M+	93	1526.88	66.70	97.21	94.91	95.66	99.08
LightAnomalyNet	7154	14	278.45	23.05	98.86	95.28	95.81	99.74

**Table 7:** Comparison of classification accuracy with the state-of-the-art methods in falling category.

	UR Fall Dataset			
Method	AUC%	Recall%	Precision%	Accuracy%
Vishnu et al. [5]	-	97.5	96.9	-
Zerrouki and Houacine [42]	-	-	-	97.0
Nunez et al. [43]	-	100.0	-	95.0
Khraief et al. [44]	-	100.0	95.0	-
LightAnomalyNet	98.71	98.92	98.79	98.86

**Table 8:** Comparison of classification accuracy with the state-of-the-art methods in suspicious actions category.

	Avenue Dataset				Mini-Drone Video			
Method	AUC%	Recall%	Precision%	Accuracy%	AUC%	Recall%	Precision%	Accuracy%
Cheoi [3]	-	94.5	93.2	90.1	-	-	-	-
Chriki et al. [52]	-	-	-	-	-	100.0	88.37	93.57
LightAnomalyNet	94.97	95.69	94.91	95.28	96.11	96.59	95.11	95.81

**Table 9:** Comparison of classification accuracy with the state-of-the-art methods in violence category.

	Hockey Fights			
Method	AUC%	Recall%	Precision%	Accuracy%
Roman and Chavez [45]	-	-	-	96.40
Song et al. [49]	-	-	-	99.62
Ullah et al. [46]	-	98.10	98.10	98.00
Asad et al. [13]	-	-	-	98.80
Mehmood [39]	99.76	99.82	99.59	99.71
LightAnomalyNet	99.78	99.81	99.66	99.74

## Data Availability

The datasets used in this study are publicly available from the sources cited in the paper.
